# Introducing P2P service to a B2C sharing platform: A hybrid sharing mode

**DOI:** 10.1371/journal.pone.0279615

**Published:** 2022-12-30

**Authors:** Tingting Tang

**Affiliations:** School of Management, University of Science and Technology of China, Hefei, Anhui, People’s Republic of China; Victoria University, AUSTRALIA

## Abstract

With the rapid development of global technology, many firms have entered the product-sharing market, where business-to-consumer (B2C), peer-to-peer (P2P), and hybrid rental services are the three most common business models. The difference between these models lies in the product provider. The product provider in B2C mode is enterprises, while in P2P mode, individuals provide the products, and both enterprises and individuals provide the products in hybrid mode. We employ a game model to analyze the influence of a B2C platform introducing P2P sharing service by comparing the profit of the platform and the original equipment manufacturer (OEM) under each of the three models. Our findings indicate that introducing P2P service always benefits the B2C platform but sometimes harms the OEM. Our findings also indicate that as the quality of the platform’s products improves, the profit improvement brought to the B2C platform by introducing P2P sharing decreases. We find that the impact of introducing P2P service also varies with consumer behaviors. For example, with increasing consumer usage level, the B2C platform’s profit improvement brought by introducing P2P sharing increases. Furthermore, we determine the optimal pricing strategies of the OEM and sharing platform in B2C mode and in hybrid mode.

## 1. Introduction

As sustainability becomes more important, consumers have explored more effective ways of using resources and products. Collaborative consumption is one way to improve sustainability. In the context of consumer-owned products not being fully used, an increasing number of platforms are entering the product-sharing market. The sharing economy can help consumers who own products obtain a portion of the benefits through sharing transactions when the products are underutilized, while consumers who do not own products trade to meet their own needs. Many sharing models do not allow changes in the commodity’s ownership; they only share the commodity’s usage right. The sharing economy prioritizes usage rights over ownership.

As the sharing economy increasingly grows, platforms have adopted various business models, among which the three most common are B2C, P2P, and hybrid rental services. (1) In the Business-to-Consumer (B2C) mode (e.g., Hello-bike), merchants or enterprises directly provide services or items to users. (2) In the Peer-to-Peer (P2P) mode (e.g., Uber), the rental products are owned by the platform’s individual product owners, and the renters are the platform’s individual users. (3) In the Hybrid mode (e.g., GoFun), both enterprises and individual owners provide services or items to users. Due to the common use of all three models, it is worthwhile to study how these sharing business models impact the market.

GoFun was founded as a B2C car rental sharing platform by the Shouqi Group, offering car rental services using cars provided by Shouqi. In September 2019, it introduced its P2P sharing service, which means the rental cars could also be provided by buyers of Shouqi Automobiles. With this change, GoFun transformed into a hybrid mode platform in which the rental cars come from both car companies and private car owners. Along with the sharing model change that Gofun has adopted came an adjustment to its rental car prices, illustrating that changes in the sharing mode will bring about changes in pricing strategies. We are curious about why GoFun introduced the P2P service and what impact the introduction had on GoFun, the Shouqi Group, and consumers. We investigate the following questions in this paper: (1) How does introducing a P2P sharing service affect a B2C sharing platform? (2) What factors influence a B2C sharing platform’s introduction of P2P sharing service, such as consumer preferences regarding rent and product quality? (3) What are the optimal pricing strategies for the OEM and platform in B2C mode and in hybrid mode?

Our investigation yielded interesting results. Introducing P2P service always benefits the B2C platform but sometimes harms the OEM. When consumer usage is high, the introduction of P2P sharing service will harm the OEM’s profits. The OEM’s profit margin increases when the product’s competition is greater because introducing P2P sharing will increase the rental utility for purchasing consumers, and it also improves the demand for the OEM’s product. The lower the quality of the platform’s products, the lower the OEM’s profit margin. This happens because P2P sharing encroaches on the demand for purchasing. The increase in the platform profit margin occurs when the platform’s product advantage decreases. On the contrary, the platform profit margin decreases when the platform’s product advantage increases.

Overall, our paper makes three contributions. First, we introduce P2P sharing service to the B2C sharing platform and employ a game model to analyze the consequences. As shown in later sections, this model considers the consumer usage level and rental discount. Second, we show that when the usage level is not too high, both the OEM and platform can get higher profits in hybrid mode. Finally, this paper contrasts the B2C mode with the hybrid mode by considering changes in the participants’ profits.

## 2. Literature review

Numerous theoretical studies have focused on the sharing economy [1–3]. Our paper relates to three streams of the sharing economy research: development and benefits, operation models, and pricing strategies (Table 1). Our goal is to highlight the significance of our work by reviewing, summarizing, and analyzing previous research efforts.

**Table 1:** Contributions and differences from previous literature.

Category	Contribution of our work	Other Literature
Development and benefits of the sharing economy	Studies a sharing scenario consisting of an OEM and a sharing platform with B2C and hybrid modes.	Discusses the challenges and opportunities presented by the sharing economy from both macro and micro perspectives.
Operation models in the sharing economy	Studies the pricing strategy for a B2C platform introducing P2P sharing service.	Examines issues under the sharing economy operating model from different perspectives.
Pricing strategies	Studies the situation assuming rental and purchase options in the sharing economy.	Focuses on pricing strategy in different scenarios.

The first stream focuses on literature about the development and benefits of the sharing economy in various scenarios [4–6]. After Felson and Spaeth [7] proposed the concept of the sharing economy, interest in it spread to all aspects of society, and it attracted increasing attention among scholars. Some scholars have used empirical methods to conduct research, such as Sundararajan [8], who considered how this new model benefits the future of economic growth. Others used modeling methods to study, such as Jiang and Tian [9], who found that transaction costs in sharing markets had nonmonotonic effects on firm profits. The characteristics of the sharing economy were also described by scholars at the beginning of its development. Botsman and Rogers [10] considered the sharing economy to have three characteristics: the acquisition of products or services without owning the assets, the redistribution of goods, and the exchange of intangible assets. Based on these characteristics, Acquier et al. [11] elaborated on the contradictory and controversial nature of the sharing economy, providing an organizational framework with three basic cores. They demonstrated how each core has different promises and paradoxes.

In addition to empirical research methods, scholars use mathematical modeling methods to conduct research in this field and investigate the impact on ownership, usage, social welfare, and sharing platforms. Focusing on consumer behavior, Benjaafar et al. [12] solved the supply and demand matching problem in a two-sided market. Xie and Sun analyzed the impact of different service stages on customer satisfaction by characterizing perceived quality [13]. Focusing on service, studies have researched cloud service [14], investigated secure m-services [15], and designed a generic Internet of Things architecture for scalable service cooperation [16]. Recommendation services for privacy-preserving tasks is also studied [17, 18]. Some studies used an intergenerational overlap model to analyze product pricing in the presence or absence of sharing. For example, Weber [19] modeled the impact on ownership demand, product prices, and returns of all participants, and Feng et al. [20] investigated the effects on luxury brands. The preceding literature discusses the challenges and opportunities presented by the sharing economy from both macro and micro perspectives, as well as the impact on society, enterprise production, and consumption, which reflects the sharing economy’s broad development prospects and academic research prospects. In our work, we study a sharing scenario consisting of an OEM and a sharing platform with B2C and hybrid modes.

The second stream explores operation models in the sharing economy, such as B2C mode, P2P mode, and hybrid mode [21, 22]. Abhishek et al. [23] firstly investigated the interaction of rental markets and OEMs in the P2P mode under alternative market structures. They focused on OEM pricing in the sharing economy from the standpoint of consumer usage heterogeneity decision-making and the business model choice of the manufacturer. Studying social welfare, Jiang and Tian [24] conducted extensive research investigating the impact of sharing platforms on manufacturers, consumers, and social welfare. Furthermore, Tian and Jiang [25] analyzed the issue from a channel perspective, arguing that P2P product sharing benefits retailers more than manufacturers. Tian et al. [26] also considered the entry of a manufacturer into the product sharing market. According to this paper, the production cost and C2C (Customer-to-Customer) shared transaction cost are the most important factors in determining the optimal usage quantity. Similarly, Wang et al. [27] characterized consumer heterogeneity along two dimensions—consumer usage level and product value preference—to study the impact of P2P sharing on enterprise participation in product sharing and enterprise product quality strategic decision-making.

Then, Bardhi and Eckhardt [28] conducted empirical research on Zipcar consumers, arguing that the sharing platform is critical to the sharing economy model. Later, scholars considered the competitive environment. For example, Guo et al. [29] empirically evaluated the impact of market entry of car-hailing platforms on new car purchases in the presence of platform competition. Additionally, Bryan and Gans [30] examined competition among ride-sharing platforms that compete on price and wait times induced by idle drivers. These articles examine issues under the sharing economy operating model from different perspectives. However, we study the pricing strategy on a B2C platform that has introduced a P2P sharing service.

The third stream addresses the specific topic of pricing strategies. This topic has gained extensive attention recently, including research on static and dynamic pricing strategies in competitive scenarios [31–36]. Xie and Sirbu [37] firstly studied incumbents’ and latecomers’ dynamic pricing behavior. Considering competition as well, Alptekinoğlu and Corbett [38] studied the competition between two multiproduct companies (mass customization and mass production) with different production technology. Similarly, scholars have studied markets in which customers have different preferences for product attributes. For example, Mendelson and Parlaktürk [39] focused on the competition of prices and product types between traditional manufacturing enterprises and personalized customization manufacturing enterprises. They analyzed the characteristics of duopoly competition between them and compared it with monopolistic competition.

Based on consumer preference, Porteus et al. [40] studied three scenarios: high-to-low customer arrival order (the later customers arrive, the lower their willingness to pay), independent order (customers’ arrival times are independent of their willingness to pay), and low-to-high customer arrival order (the later customers arrive, the higher their willingness to pay). They analyzed product differentiation competition and pricing decision problems for leaders and followers in the market. Also considering different products, Subramaniam and Gal-Or [41] extended the standard Hoteling model of product differentiation. They focused on the problem of quantity discounting in differentiated consumer product markets. Similarly, Nasser and Turcic [42] used the Hoteling model as a benchmark to analyze a multistage game under a duopoly from the perspective of commitment decision-making. The symmetrical equilibrium allows two companies to avoid the intense price competition associated with low product differentiation. For asymmetric firms, Wu and Lai [43] considered price competition in a multistage game. This research shows that launching new products later than competitors is often a dominant strategy for companies. The above studies all focus on pricing strategy in different scenarios, whereas we study the situation under the rental and purchase options of the sharing economy.

Our research analyzes why a B2C sharing platform introduces P2P sharing service. In other words, why will a sharing platform adopt the hybrid sharing mode of including B2C and P2P sharing service? This paper differs from other research in the following ways. First, we analyze the B2C sharing mode, and we concentrate on the platform rather than the OEM. Second, we explore the optimal pricing decisions of the platform and OEM in B2C sharing mode and hybrid sharing mode. Third, in an extension, we further consider the situation of the platform deciding the rental price. This broadens the applicability of our management discoveries.

## 3. Model and analysis

The market contains one B2C sharing platform (labeled *P*). It has its own product (Product 2) for sharing-rental, the quality of which is λ(λ∈(0,1)). A competing OEM (labeled *O*) is also in this market, and it produces Product 1 for selling, the quality of which is normalized to 1. A unit of Product 1 produced by the OEM costs *c*_1_. The cost of the sharing platform to own its unit product is *c*_2_. We assume that *c*_1_, *c*_2_∈(0,1).

We consider a population of consumers who are heterogeneous in their product quality preference *θ*(*θ*~*U*[0,1]). The total number of consumers is normalized to 1. We use *u*(*u*∈(0,1)) to describe the product’s use frequency per consumer, also called the usage level, and we assume it is homogeneous. When a consumer (she) rents a product, she will have a discount *β*(*β*∈(0,1)) on the rental product’s quality. Factors such as her lack of ownership of the product and the inconvenience of using the rental product will make her perceive the quality of the product at a discount compared to buying the product. This means her valuation of renting a product is lower than her valuation of buying the same product.

In the case of no P2P sharing service (case *N*), a consumer who wants to use the product has two choices: one is spending *p* to buy (*B*) a Product 1 from the OEM, and the other is spending *r*_2_ to rent (*R*) a Product 2 from the B2C sharing platform.

In the presence of P2P sharing service (case *P*), a consumer who wants to use the product has one more choice: using *r*_1_ to rent a Product 1 from the sharing platform with P2P sharing. The model of Jiang and Tian [24] assumes that demand and supply are balanced in the P2P sharing market through a market-clearing price, as does our model. The rent is *r*_1_ per unit, and that is decided with no matching friction. Typically, the platform sets a commission *t* as a service charge, where *t*<*r*_1_, so that owners can get *r*_1_−*t* income by renting out one unit of usage. This paper’s key notation is summarized in Table 2.

**Table 2:** Summary of notation.

Notation	Description
*i* = *B*, *R*	Buy or Rent
*j* = 1,2	Product 1 or Product 2
*m* = *N*, *P*, *D*, *E*	Case *N* or case *P* or case *D* or case *E*
*n* = *O*, *P*	OEM or Platform
*θ*	Consumer’s product quality preference, *θ*~*U*[0,1]
*u*	Product usage level, *u*∈(0,1)
*β*	Consumer value discounts on the quality of rental products, *β*∈(0,1)
*λ*	Quality of platform’s own products, *λ*∈(0,1)
*p*	Price of OEM’s product
*r*_1_	OEM’s product sharing price (rental price)
*r*_2_	Platform’s own product sharing price (rental price)
*t*	Transaction service fee for each unit rent out of the platform’s own products
*c*_1_	Cost of OEM’s product, *c*_1_∈(0,1)
*c*_2_	Cost of platform’s product, *c*_2_∈(0,1)
$U_{j}^{mi}$	Consumer’s utility of *i* Product *j* in case *m*
$D_{j}^{mi}$	Consumer’s demand of *i* Product *j* in case *m*
$\Pi_{n}^{m}$	Profit of *n* in case *m*
*	Equilibrium (optimal) results

### 3.1 Benchmark: B2C sharing scenario (case *N*)

**Fig 1:**
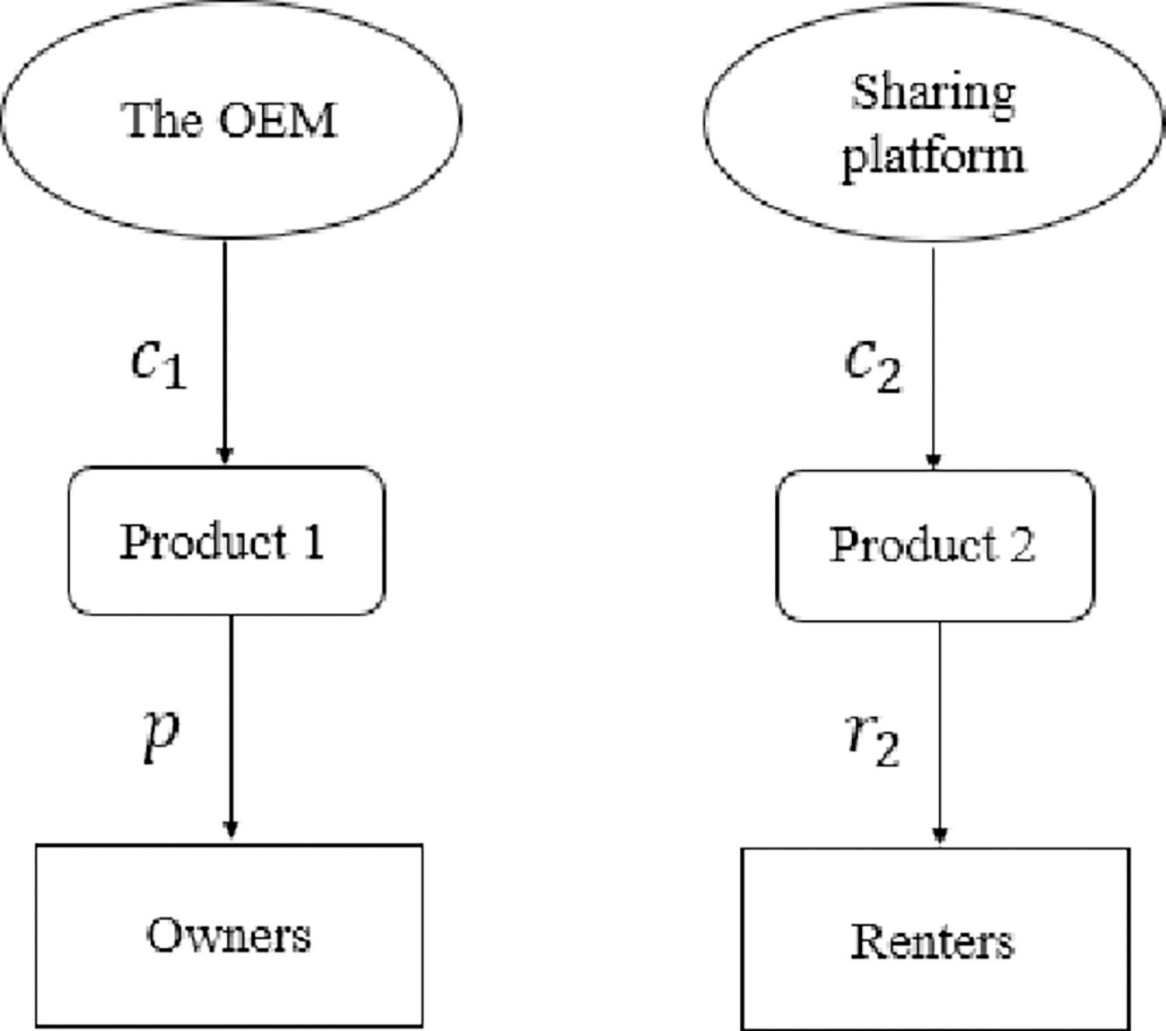
Model structure of benchmark.

In this section, we consider case *N*, in which the platform only uses the B2C sharing mode (Fig 1). We assume that the platform provides unlimited products to meet all needs. When the consumer buys Product 1, her utility can be expressed as $U_{1}^{NB}=u\theta -p$. For renting Product 2, her utility is $U_{2}^{NR}=u(\beta \theta \lambda -r_{2})$. For choosing the outside option, her utility is normalized to 0.

Based on the parameter *θ*, the population can be segmented into owners, renters, and neither. By considering $U_{1}^{NB}>U_{2}^{NR}$ and $U_{1}^{NB}>0$, we can determine that the owners will be the consumers with $\theta >\frac{p-ur_{2}}{u(1-\beta \lambda )}$. By considering $U_{1}^{NB}<U_{2}^{NR}$ and $U_{2}^{NR}>0$, we can get the renters will be the consumers with $\frac{p-ur_{2}}{u(1-\beta \lambda )}>\theta >\frac{r_{2}}{\beta \lambda }$, and the rest of the consumers choose an external option. Therefore, the demand to own Product 1 is $D_{1}^{NB}=1-\frac{p-ur_{2}}{u(1-\beta \lambda )}$, and the demand for renting Product 2 is $D_{2}^{NR}=\frac{p-ur_{2}}{u(1-\beta \lambda )}-\frac{r_{2}}{\beta \lambda }$.

In this basic model, the question is how the OEM should optimally price its products. The profit of the OEM is $\Pi_{O}^{N}=(p-c_{1})(1-\frac{p-ur_{2}}{u(1-\beta \lambda )})$. The platform profit is $\Pi_{P}^{N}=(r_{2}-c_{2})(\frac{p-ur_{2}}{u(1-\beta \lambda )}-\frac{r_{2}}{\beta \lambda })$.

#### Lemma 1

In case N, when the OEM and platform are in the B2C sharing scenario, the OEM’s optimal selling price *p*^N*^, the demands $D_{1}^{NB*}$ and $D_{2}^{NR*}$, the profit of the OEM $\Pi_{O}^{N*}$, and the profit of the platform $\Pi_{P}^{N*}$ are as shown in Table 3.

**Table 3:** Equilibrium results in B2C sharing scenario (case *N*).

Variable	Value
*p* ^N*^	$\frac{c_{1}+u(1-\beta \lambda +r_{2})}{2}$
$D_{1}^{NB*}$	$\frac{u-u\beta \lambda -c_{1}+ur_{2}}{2u(1-\beta \lambda )}$
$D_{2}^{NR*}$	$\frac{\beta \lambda (u-u\beta \lambda +c_{1})+u(-2+\beta \lambda )r_{2}}{2u\beta \lambda (1-\beta \lambda )}$
$\Pi_{O}^{N*}$	$\frac{{\left(c_{1}+u(-1+\beta \lambda -r_{2})\right)}^{2}}{4u(1-\beta \lambda )}$
$\Pi_{P}^{N*}$	$\frac{\left(r_{2}-c_{2})(\beta \lambda (u-u\beta \lambda +c_{1})+u(-2+\beta \lambda )r_{2}\right)}{2u\beta \lambda (1-\beta \lambda )}$

The proofs of all lemmas and propositions in this paper are provided in the S1 Appendix. We analyze the results in the equilibrium solution in Section 4.

### 3.2 Hybrid sharing scenario (case *P*)

**Fig 2:**
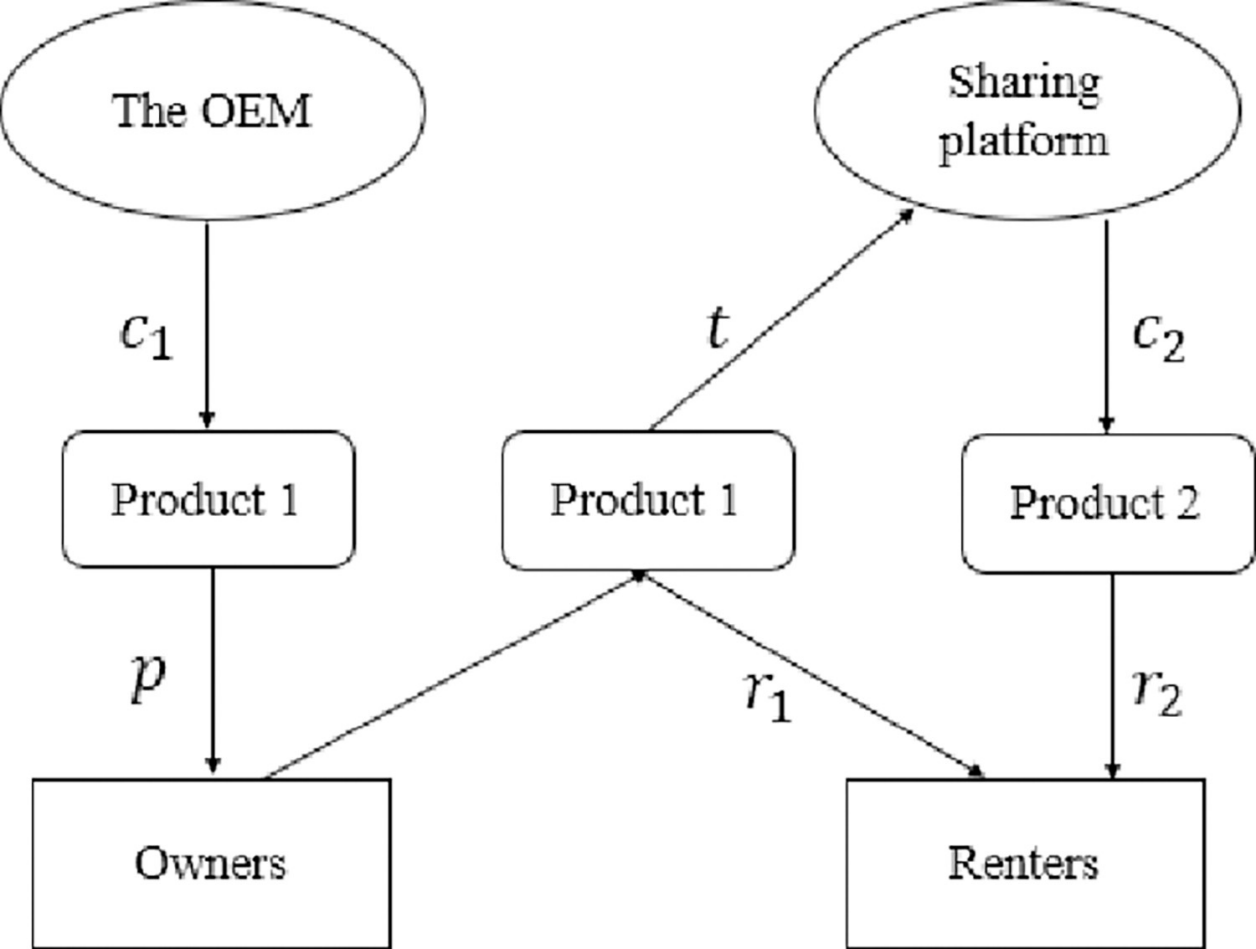
Model structure of hybrid sharing scenario.

We have analyzed the prices, demands, and equilibrium outcomes for the benchmark case *N*. In this section, we consider case *P*, where owners rent their products through the platform when they are not using them (Fig 2). In case *P*, we study how P2P sharing affects the two companies’ profits. We assume that the platform product’s sharing price *r*_2_ is exogenously determined to focus on the optimal price of Product 1. In Section 4, we will discuss the situation when *r*_2_ is endogenous.

The consumer’s utility of buying Product 1 can be expressed as $U_{1}^{B}=u\theta +(1-u)(r_{1}-t)-p$. If she rents Product 1, her utility is $U_{1}^{R}=u(\beta \theta -r_{1})$.

Based on the parameter *θ*, the population can be segmented in equilibrium: By considering $U_{1}^{B}>U_{1}^{R}$ and $U_{1}^{B}>0$, we find that consumers with $\theta >\frac{p+t(1-u)-r_{1}}{u(1-\beta )}$ are owners. Considering $U_{1}^{R}>U_{2}^{R}$ and $U_{1}^{R}>U_{1}^{B}$ shows that consumers with $\frac{p+t(1-u)-r_{1}}{u(1-\beta )}>\theta >\frac{r_{1}-r_{2}}{\beta (1-\lambda )}$ are renters for Product 1. Considering $U_{2}^{R}>U_{1}^{R}$ and $U_{2}^{B}>0$ shows that consumers with $\frac{r_{1}-r_{2}}{\beta (1-\lambda )}>\theta >\frac{r_{2}}{\beta \lambda }$ are renters for Product 2. The remaining consumers choose external options.

We use *P* to indicate the market with P2P sharing service. The profit of the OEM is $\Pi_{O}^{P}=(p-c_{1})(1-\frac{p+t(1-u)-r_{1}}{u(1-\beta )})$. The platform profit is $\Pi_{P}^{P}=tu(\frac{p+t(1-u)-r_{1}}{u(1-\beta )}-\frac{r_{1}-r_{2}}{\beta (1-\lambda )})+(r_{2}-c_{2})(\frac{r_{1}-r_{2}}{\beta (1-\lambda )}-\frac{r_{2}}{\beta \lambda })$.

By matching the supply and demand of Product 1 $(1-u)D_{1}^{B}=uD_{1}^{R}$, we can obtain the sharing market clearing price: $r_{1}(p,t)=\frac{(p-(-1+u)(t+u(-1+\beta )))\beta (-1+\lambda )+u^{2}(-1+\beta )r_{2}}{u^{2}(-1+\beta )+\beta (-1+\lambda )}$.

To solve this optimization problem, we can substitute *r*_1_(*p*, *t*) into the above formulas and consider the first-order condition with respect to *p* and *t*. This allows us to calculate the profits and optimal prices of both parties.

#### Lemma 2

In case P, when the OEM and platform are in the hybrid sharing scenario, the OEM’s optimal selling price *p*^P*^, the OEM’s optimal retail price $r_{1}^{P*}$, the platform’s optimal service charge *t*^*P*^, the demands ($D_{1}^{PB*},\;D_{1}^{PR*},\;and\;D_{2}^{PR*}$), the OEM profit $\Pi_{O}^{P*}$, and the platform profit $\Pi_{P}^{P*}$ are as shown in Table 4.

**Table 4:** Equilibrium results in hybrid sharing scenario (case *P*).

Variable	Value
*p* ^P*^	$\frac{u(u+\beta -u\beta -\beta \lambda )+2uc_{1}+c_{2}+(-1+u)r_{2}}{3u}$
*t* ^P*^	$\frac{u(u(-1+\beta )+\beta (-1+\lambda ))+uc_{1}+2c_{2}-(2+u)r_{2}}{3(-1+u)u}$
$r_{1}^{P*}$	$\frac{-\beta (-1+\lambda )(u(u(-1+3u)(-1+\beta )-2\beta +2\beta \lambda -c_{1})+c_{2})+(3u^{3}(-1+\beta )+\beta (-1+\lambda )+2u\beta (-1+\lambda ))r_{2}}{3(u^{3}(-1+\beta )+u\beta (-1+\lambda ))}$
$D_{1}^{PB*}$	$\frac{u(u(-1+\beta )+\beta (-1+\lambda ))+uc_{1}-c_{2}-(-1+u)r_{2}}{3(u^{2}(-1+\beta )+\beta (-1+\lambda ))}$
$D_{1}^{PR*}$	$\frac{\left(-1+u)(u(u(-1+\beta )+\beta (-1+\lambda ))+uc_{1}-c_{2}-(-1+u)r_{2}\right)}{3(u^{3}(1-\beta )+u\beta (1-\lambda ))}$
$D_{2}^{PR*}$	$\frac{\beta \lambda (u(u(-1+3u)(-1+\beta )-2\beta +2\beta \lambda -c_{1})+c_{2})-(3u^{3}(-1+\beta )+\beta \lambda +u\beta (-3+2\lambda ))r_{2}}{3u\beta (u^{2}(-1+\beta )+\beta (-1+\lambda ))\lambda }$
$\Pi_{O}^{P*}$	$\frac{{\left(u(u+\beta -u\beta -\beta \lambda )-uc_{1}+c_{2}+(-1+u)r_{2}\right)}^{2}}{9(u^{3}(1-\beta )+u\beta (1-\lambda ))}$
$\Pi_{P}^{P*}$	$\left((-u^{2}\beta \lambda {\left(u+\beta -u\beta -\beta \lambda \right)}^{2}-(9u^{3}(-1+\beta )-9u\beta +(1+u(7+u))\beta \lambda ){r_{2}}^{2}+(9u^{3}(-1+\beta )+2\beta \lambda +u\beta (-9+7\lambda ))c_{2}+\beta \lambda (-u^{2}c_{1}(2u(-1+\beta )+2\beta (-1+\lambda )+c_{1})+u(-u(-2+9u)(-1+\beta )+7\beta -7\beta \lambda +2c_{1})c_{2}-{c_{2}}^{2})+(u\beta \lambda (11u^{2}(-1+\beta )+2u(1+\beta (-2+\lambda ))+7\beta (-1+\lambda )+2(-1+u)c_{1}))/(9u\beta (u^{2}(-1+\beta )+\beta (-1+\lambda ))\lambda \right)$

Lemma 2 shows that equilibrium solutions for both P2P sharing and B2C sharing exist. We analyze the results under the equilibrium solution in Section 4.

We can substitute the equilibrium solution back into the profit functions, and the result is a high-order analytical formula that is difficult to analyze theoretically. Therefore, to obtain management inspiration, we use numerical analysis. The following subsection compares the supply chain with and without P2P sharing service, and we analyze the impact on the B2C platform and OEM.

## 4. Discussion and numerical analysis

In this section, we firstly analyze the results under the above two equilibrium solutions. Then, we examine the impact of product sharing on the OEM profit and platform profit.

**Proposition 1.** *In case N*, *when the OEM and platform are in the B2C sharing scenario*:

1. $p^{N*},\;D_{1}^{NB*}$, and $\Pi_{O}^{N*}$ increase in u, whereas $D_{2}^{NR*}$ and $\Pi_{P}^{N*}$ *decrease in u.*
2. p^N*^ decreases in β and in λ. When c_1_<ur_2_, $D_{1}^{NB*}$ *increases in β and in λ; when c_1_>ur_2_*, $D_{1}^{NB*}$ *decreases in β and in λ. When* $c_{1}>\frac{-u(2+\beta \lambda (-4+\beta \lambda ))r_{2}}{\beta^{2}\lambda^{2}},\;D_{2}^{NR*}$ *and* $\Pi_{P}^{N*}$ *increase in β and in λ; when* $c_{1}<\frac{-u(2+\beta \lambda (-4+\beta \lambda ))r_{2}}{\beta^{2}\lambda^{2}},\;D_{2}^{NR*}$ *and* $\Pi_{P}^{N*}$ *decrease in β and in λ. When c_1_>u(r_2_+1−βλ) or c_1_<u(r_2_−1+βλ)*, $\Pi_{O}^{N*}$ *increases in β and in λ; when u(r_2_−1+βλ)<c_1_<u(r_2_+1−βλ)*, $\Pi_{O}^{N*}$ *decreases in β and in λ*.

In a two-player supply chain, the impact of the OEM decision-making on a supply chain using P2P mode has been shown in the literature. Whether the same conclusions hold when the platform can make the decisions is not obvious, but our research will unveil these results.

Proposition 1 (1) shows that when the usage level u increases, the OEM profit rises in the B2C sharing mode. This happens because both the Product 1 price *p*^N*^ and the demand for owning it $D_{1}^{NB*}$ increase as the usage level increases. Moreover, the demand for renting Product 2 $D_{2}^{NR*}$ and the platform profit $\Pi_{P}^{N*}$ decrease in the usage level *u*.

Proposition 1 (2) shows that depending on the quality of Product 2 *λ* and the rental discount *β*, B2C sharing can result in either lower or higher OEM profit $\Pi_{O}^{N*}$ and platform profit $\Pi_{P}^{N*}$. When the cost of Product 1 *c*_1_ is sufficiently low, the platform’s profit decreases when either *β* or *λ* increases. When the cost of Product 1 *c*_1_ is moderate, the profit of the OEM $\Pi_{O}^{N*}$ decreases with increasing *β* or *λ*. Moreover, the price of Product 1 *p*^N*^ is decreasing in both *λ* and *β*. The fact that the ownership of Product 1 $D_{1}^{NB*}$ increases or decreases in the quality of Product 2 *λ* and the rental discount *β* is perhaps surprising. It shows that an increase in the value of renting Product 2 (*λ* or *β*) is likely to lead to less demand for this product $D_{1}^{NB*}$ when the cost of Product 1 *c*_1_ is high. This can be explained as follows. The increase in Product 2’s quality *λ* will intensify the competition between the two products, so the OEM’s equilibrium price *p*^N*^ will be set lower to increase competitiveness. When the cost is high, having only the usage of Product 2 increase is nonsense because increased usage through buying Product 1 leads to less demand for Product 2 $D_{2}^{NR*}$. In contrast, when the cost is low, the competitiveness of Product 1 can be increased by price reduction, but Product 2 achieves no better competitiveness by improving usage value (quality *λ* or rental discount *β*). In the following, we discuss the question of whether hybrid sharing reduces profits.

**Proposition 2.** *In case P*, *when the OEM and platform are in the hybrid sharing scenario*:

1. *p*^P*^ *increases in u. When* $c_{1}<\frac{\left(4u-2)(r_{2}-c_{2}\right)}{u^{2}}+1-\beta \lambda +r_{2},\;t^{P*}$ *increases in u; when* $c_{1}>\frac{\left(4u-2)(r_{2}-c_{2}\right)}{u^{2}}+1-\beta \lambda +r_{2},\;t^{P*}$ *decreases in u.*
2. *When u*<1−*λ*, *p*^P*^ *and t*^P*^ *increase in β; when u*>1−*λ*, *p*^P*^ *and t*^P*^ *decrease in β*.
3. *p*^P*^ *and t*^P*^ *decrease in λ*.

Proposition 2 (1) shows how changes in the exogenous parameter impact the decision variables under hybrid sharing. As the usage level *u* increases, the price of Product 1 *p*^P*^ increases. Depending on the cost of Product 1 *c*_1_, the commission of P2P sharing *t*^P*^ can be either lower or higher. When the cost *c*_1_ is sufficiently high, the commission *t*^P*^ decreases with increasing *u*.

Proposition 2 (2) shows that when the usage level *u* is sufficiently high, the price of Product 1 *p*^P*^ and the commission *t*^P*^ decrease when the rental discount *β* decreases. In this case, the lower usage level means lower use frequency. When the frequency of use is lower, consumers are more inclined to rent rather than buy. Also, the price of Product 1 *p*^P*^ and the commission *t*^P*^ decrease as the quality of Product 2 *λ* increases. The reason that *p*^P*^ is decreasing in *λ* can be explained as follows. The Product 2 quality increase means the Product 2 usage value increases, so the competition between Product 1 and Product 2 intensifies. To improve the competitiveness of Product 1, the OEM must lower its price to maximize profit, which is manifested as a decrease in *p*^P*^.

We substitute the equilibrium solution back to the profit functions, and each result is a high-order analytical formula, which is difficult to analyze theoretically. Therefore, to obtain management insights, we use numerical analysis.

**Fig 3:**
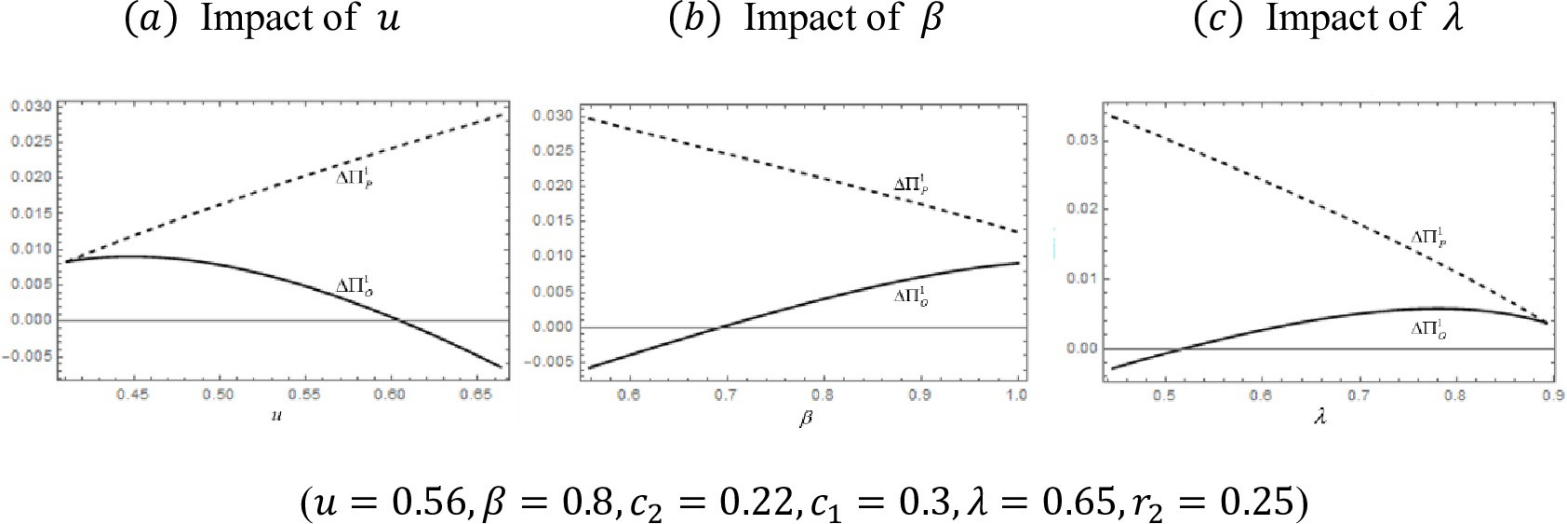
Impact of exogenous variables on $\Delta \Pi_{O}^{1}\;and\;\Delta \Pi_{P}^{1}.$. (*a*) Impact of *u*, (*b*) Impact of *β*, (*c*) Impact of *λ*.

Let $\Delta \Pi_{O}^{1}$ denote the difference in the OEM profits $\Pi_{O}^{P*}-\Pi_{O}^{N*}$. We denote the difference in platform profits $\Pi_{P}^{P*}-\Pi_{P}^{N*}$ as $\Delta \Pi_{P}^{1}$. Fig 3 summarizes the profit comparison under different exogenous parameters.

Fig 3 depicts the profit gaps (between hybrid mode and B2C mode) of the OEM and the platform as parameters *u*, *β*, and *λ* vary. When the curve is over the abscissa, the margin is positive, which is when the OEM or platform prefer the hybrid mode. The following summarizes how profits, prices, and demands under B2C mode compare to those under hybrid mode to explain the figures above.

First, we analyze the effect of usage level *u* on the profit margin, as seen in Fig 3(A). As the usage level *u* increases, the increase in profit from the platform’s P2P sharing $\Delta \Pi_{P}^{1}$ is on the rise because renting Product 1 brings more revenue to the platform. The OEM profit margin $\Delta \Pi_{O}^{1}$ decreases because P2P sharing brings more choices and encroaches on the profits of the OEM. Although the OEM’s profit is also increasing in hybrid mode, it does not increase as significantly as under B2C mode. As a result, the profit margin decreases. We can interpret the image in more depth by analyzing the change in profit of B2C mode and hybrid mode separately. Under B2C mode, as the usage level rises, the OEM profit rises and the platform profit falls because when the usage level is high, consumers prefer to own the product rather than rent. With P2P sharing, as the usage level increases, more consumers buy Product 1, which increases corporate profit. P2P sharing service brings more rentable products to the P2P rental market for Product 1, greatly increasing the platform’s service fee income.

Next, we analyze the effect of rental discount *β* on the profit margin, which is illustrated in Fig 3(B). With the improvement of rental discount *β*, the profit increase brought by the platform’s P2P sharing declines, which is equivalent to increasing the platform’s product value. P2P sharing makes the platform’s profit $\Delta \Pi_{P}^{1}$ smaller because the competition between rented and purchased products becomes more intense. The increase in the OEM profit margin $\Delta \Pi_{O}^{1}$ is due to the increase in rental income from purchased products, which promotes the purchase of products and increases the income from the sale of products. Through analysis of the platform and OEM profits in the two modes, we have a more detailed understanding: Under B2C mode, the OEM profit falls as the rental discount rises because when the platform’s product rental discount increases, the OEM’s produce price and profit decrease due to increased competition. In the presence of P2P sharing, as the rental discount increases, the OEM’s profit decreases. Also, because the competition is more intense, both demand and optimal pricing decrease. In particular, the profit of the platform fluctuates. Due to the increase in the quality perception of rental Product 2, the revenue from the demand for renting increases. However, the number of products available for rent becomes smaller, and the shrinking size of this part of the market reduces revenue.

We now proceed to analyze the effect of quality difference *λ* on the profit margin, which is illustrated in Fig 3(C). As the quality of Product 2 *λ* improves, the profit margin of the platform $\Delta \Pi_{P}^{1}$ decreases. Although the revenue of the rental business increases, the service fee revenue is reduced. The increase in the OEM profit $\Delta \Pi_{O}^{1}$ is due to the increase in rental income from purchased products, which promotes the demand for owning products and increases income from sales. Now let us conduct a more in-depth analysis of the image with the conclusions obtained in Section 3. Under B2C mode, as the difference in product quality decreases, the OEM profit decreases because the closeness of the quality intensifies the competition between the two products. In the presence of P2P sharing, the amount by which the rising Product 2 quality improves the OEM’s profit decreases because the competition is more intense. Note that the profit of the platform decreases. In particular, the revenue from Product 2 increases. Intuitively, the revenue of Product 1 is reduced due to the decrease in the number of products available for rent. The market for renting Product 1 is encroached upon by rentals of Product 2, and the final loss is not offset.

The increase in the OEM’s profit margin occurs when the product’s competition is greater because the P2P sharing will increase the rental utility for purchasing consumers, and it also improves the demand for Product 1 compared to the case of no P2P sharing. The more advantageous Product 1 is to sell, the lower the OEM’s profit margin; this happens because the P2P sharing takes away some of the market demand for purchases. The increase in the platform profit margin occurs when the platform’s own product advantage decreases. Note that the platform profit margin decreases when the platform’s own product advantage increases.

## 5. Extension: Endogenous rental price

As observed in practice, many platforms decide the rental price by themselves. In this extension, we study the situation where the platform can decide the rental price of its product. Paralleling the analysis of the main model, we first explore the results in the B2C mode (case *D*), then solve the results of adding the P2P sharing (case *E*), and finally compare the two modes.

### 5.1 B2C sharing scenario (case *D*)

In this subsection, we analyze and discuss the robustness of letting *r*_2_ be a decision variable set by the platform. The question is how the OEM can optimally price its product and how the platform should set *r*_2_. Under this scenario, the OEM decides the price of Product 1 *p*, while the platform decides the rental price of Product 2 *r*_2_.

#### Lemma 3

In case D, when the OEM and platform are in the B2C sharing scenario and the platform decides the rental price, the OEM’s optimal selling price p^D*^, platform’s optimal rental price $r_{2}^{D*}$, demands $D_{1}^{DB*}\;and\;D_{2}^{DR*}$, OEM profit $\Pi_{O}^{D*}$, and platform profit $\Pi_{P}^{D*}$ are as shown in Table 5.

**Table 5:** Equilibrium results in B2C sharing scenario with deciding *r*_2_ (case *D*).

Variable	Value
*p* ^D*^	$\frac{2c_{1}+u(2-2\beta \lambda +c_{2})}{4-\beta \lambda }$
$r_{2}^{D*}$	$\frac{u\beta \lambda (-1+\beta \lambda )-\beta \lambda c_{1}-2uc_{2}}{u(-4+\beta \lambda )}$
$D_{1}^{DB*}$	$\frac{\left(-2+\beta \lambda )c_{1}+u(2-2\beta \lambda +c_{2}\right)}{u(-4+\beta \lambda )(-1+\beta \lambda )}$
$D_{2}^{DR*}$	$\frac{\beta \lambda (u-u\beta \lambda +c_{1})+u(-2+\beta \lambda )c_{2}}{u\beta \lambda (-4+\beta \lambda )(-1+\beta \lambda )}$
$\Pi_{O}^{D*}$	$\frac{{\left(\left(-2+\beta \lambda )c_{1}+u(2-2\beta \lambda +c_{2}\right)\right)}^{2}}{u{\left(-4+\beta \lambda \right)}^{2}(1-\beta \lambda )}$
$\Pi_{P}^{D*}$	$\frac{{\left(\beta \lambda (u-u\beta \lambda +c_{1})+u(-2+\beta \lambda )c_{2}\right)}^{2}}{u^{2}\beta \lambda {\left(-4+\beta \lambda \right)}^{2}(1-\beta \lambda )}$

Lemma 3 shows that the equilibrium solution of the problem exists, and so we proceed to analyze the results under the equilibrium solution.

**Proposition 3.** *In case D*, *when the OEM and platform are in the B2C sharing scenario and the platform decides the rental price*:

1. pD*, $D_{1}^{DB*}$ *and* $\Pi_{O}^{D*}$ *increase in u, whereas* $r_{2}^{D*},\;D_{2}^{DR*}$, *and* $\Pi_{P}^{D*}$ *decrease in u.*
2. p^D*^ decreases in β and in λ. When $c_{1}>\frac{-u(4+\beta \lambda (-8+\beta \lambda )+2c_{2})}{4},\;r_{2}^{D*}$ increases in β and in λ; when $c_{1}<\frac{-u(4+\beta \lambda (-8+\beta \lambda )+2c_{2})}{4},$ r_2_* decreases in β and in λ.
3. $p^{D*},\;r_{2}^{D*},\;D_{2}^{DR*}$ and $\Pi_{P}^{D*}$ *increase in c*_1_*, whereas $D_{1}^{DB*}$ and $\Pi_{O}^{D*}$ decrease in c*_1_.
4. $p^{D*},\;r_{2}^{D*},\;D_{1}^{DB*}$ and $\Pi_{O}^{D*}$ *increase in c*_2_*, whereas* $D_{2}^{DR*}$ and $\Pi_{P}^{D*}$ *decrease in c*_2_.

Proposition 3 (1) shows that the Product 1 price *p*^D*^, demand for buying Product 1 $D_{1}^{DB*}$, and OEM profit $\Pi_{O}^{D*}$ all are increasing in the usage level *u*. In contrast, the rent for Product 2 $r_{2}^{D*}$, demand for renting Product 2 $D_{2}^{DR*}$, and platform profit $\Pi_{P}^{D*}$ are all decreasing in the usage level *u*. Compared to case *N*, where the platform cannot decide the rent of Product 2 *r*_2_, the platform can raise its profit by setting *r*_2_.

Proposition 3 (2) shows that the price of Product 1 *p*^D*^ decreases in the rental discount *β* and in Product 2’s quality *λ*. This can be explained as follows. When Product 2’s usage value increases, the OEM will increase the competitiveness by reducing Product 1’s selling price to obtain the optimal profit at equilibrium. Depending on the Product 1 cost *c*_1_, the rental price $r_{2}^{D*}$ can increase or decrease with the increase in the rental discount *β* and the Product 2 quality *λ*. When the cost is sufficiently low, the fact that $r_{2}^{D*}$ is increasing in *β* and in *λ* is perhaps surprising as it shows that an increase in the product’s usage value can reduce its rental cost. This is caused by changes in the demand for the product itself $D_{2}^{DR*}$ and for its competitor $D_{1}^{DB*}$. The pricing of a product is not only affected by the product itself but also by its demand. The following subsection discusses whether it is optimal to add P2P sharing in case *D*.

Proposition 3 (3) shows that the Product 1 price *p*^D*^, rent for Product 2 $r_{2}^{D*}$, demand for renting Product 2 $D_{2}^{DR*}$, and platform profit $\Pi_{P}^{D*}$ are all increasing in Product 1’s cost *c*_1_. In contrast, the demand for buying Product 1 $D_{1}^{DB*}$ and the OEM profit $\Pi_{O}^{D*}$ are decreasing in Product 1’s cost *c*_1_.

Proposition 3 (4) shows that the Product 1 price *p*^D*^, rent for Product 2 $r_{2}^{D*}$, demand for buying Product 1 $D_{1}^{DB*}$ and OEM profit $\Pi_{O}^{D*}$ are all increasing in Product 2’s cost *c*_2_. In contrast, the demand for renting Product 2 $D_{2}^{DR*}$ and the platform profit $\Pi_{P}^{D*}$ are decreasing in Product 2’s cost *c*_2_.

### 5.2 Hybrid sharing scenario (case *E*)

In this subsection, we consider how P2P sharing in the B2C sharing mode impacts the equilibrium results with endogenous product rental price decisions. Our profit function and consumer utility are expressed in the same way as in Section 3.2. In this scenario, the OEM decides Product 1’s price *p*, while the platform decides both the rental price of Product 2 *r*_2_ and the transaction service fee *t* at the same time. By calculation, we can get the following lemma.

#### Lemma 4

In case E, when the OEM and platform are in the hybrid sharing scenario and the platform decides the rental price, the OEM’s optimal selling price *p*^E*^; OEM’s optimal retail price $r_{1}^{E*}$; platform’s optimal service charge *t*^E*^; platform’s optimal rent $r_{2}^{E*}$; demands $D_{1}^{EB*},\;D_{1}^{ER*},\;and\;D_{2}^{ER*}$; OEM profit $\Pi_{O}^{E*}$; and platform profit $\Pi_{P}^{E*}$ are as shown in Table 6.

**Table 6:** Equilibrium results in Hybrid sharing scenario with deciding *r*_2_ (case *E*).

Variable	Value
*p* ^E*^	$\frac{\left(4u^{3}(-1+\beta )-4u\beta +{\left(1+u\right)}^{2}\beta \lambda )c_{1}-(u^{2}(-1+\beta )+\beta (-1+\lambda ))(2u^{2}(-1+\beta )+u\beta (-2+\lambda )+\beta \lambda -(1+u)c_{2}\right)}{6u^{3}(-1+\beta )-6u\beta +(1+u(4+u))\beta \lambda }$
*t* ^E*^	$\frac{2u(u^{2}(-1+\beta )-\beta )(-u+(-1+u)\beta )+\beta (u(-1+(-1+u)u(-1+\beta )-\beta )+\beta )\lambda -\beta^{2}\lambda^{2}+(2u^{3}(-1+\beta )+u\beta (-2+\lambda )+\beta \lambda )c_{1}+(-(-2+u)(u^{2}(-1+\beta )-\beta )+\beta \lambda )c_{2}}{\left(-1+u)(6u^{3}(-1+\beta )-6u\beta +(1+u(4+u))\beta \lambda \right)}$
$r_{2}^{E*}$	$\frac{u\beta \lambda (u+4u^{2}(-1+\beta )+u\beta (-2+\lambda )+2\beta (-1+\lambda )+(-1+u)c_{1})+(3u^{3}(-1+\beta )+\beta \lambda +u\beta (-3+2\lambda ))c_{2}}{6u^{3}(-1+\beta )-6u\beta +(1+u(4+u))\beta \lambda }$
$r_{1}^{E*}$	$\frac{u\beta (-2u^{2}(-1+\beta )(-3+\lambda )+u(-1+\beta )(-2+\lambda )-\beta (-4+\lambda )(-1+\lambda )+(-2+\lambda +u\lambda )c_{1})+(3u^{3}(-1+\beta )+\beta +u\beta (-2+\lambda ))c_{2}}{6u^{3}(-1+\beta )-6u\beta +(1+u(4+u))\beta \lambda }$
$D_{1}^{EB*}$	$\frac{u(2u^{2}(-1+\beta )+u\beta (-2+\lambda )+\beta \lambda +2uc_{1}-(1+u)c_{2})}{6u^{3}(-1+\beta )-6u\beta +(1+u(4+u))\beta \lambda }$
$D_{1}^{ER*}$	$\frac{-(-1+u)(2u^{2}(-1+\beta )+u\beta (-2+\lambda )+\beta \lambda +2uc_{1})+(-1+u^{2})c_{2}}{6u^{3}(-1+\beta )-6u\beta +(1+u(4+u))\beta \lambda }$
$D_{2}^{ER*}$	$\frac{u\beta \lambda (u+2u^{2}(-1+\beta )+\beta (-2+\lambda )-(1+u)c_{1})+u(-3u^{2}(-1+\beta )-\beta (-3+\lambda ))c_{2}}{\beta \lambda (6u^{3}(-1+\beta )-6u\beta +(1+u(4+u))\beta \lambda )}$
$\Pi_{O}^{E*}$	$\frac{u(u^{2}(1-\beta )+\beta (1-\lambda )){\left(2u^{2}(-1+\beta )+u\beta (-2+\lambda )+\beta \lambda +2uc_{1}-(1+u)c_{2}\right)}^{2}}{{\left(6u^{3}(-1+\beta )-6u\beta +(1+u(4+u))\beta \lambda \right)}^{2}}$
$\Pi_{P}^{E*}$	$\left(u(\beta \lambda (-4u^{2}(u^{2}(-1+\beta )-\beta ){\left(u+\beta -u\beta \right)}^{2}+u\beta (6u^{3}(-1+\beta )+4u^{4}{\left(-1+\beta \right)}^{2}-6u\beta +4\beta^{2}+u^{2}(-1-8(-1+\beta )\beta ))\lambda +\beta^{2}(-\beta +u(1-8\beta +u(1-u(7+u)+u(8+u)\beta )))\lambda^{2}+(1+u(3+u))\beta^{3}\lambda^{3})-u\beta \lambda (4u^{3}(-1+\beta )-4u\beta +{\left(1+u\right)}^{2}\beta \lambda )c_{1}^{2}-\beta \lambda (4u^{3}(-1+\beta )-4u\beta +{\left(1+u\right)}^{2}\beta \lambda )c_{1}(2u^{2}(-1+\beta )+u\beta (-2+\lambda )+\beta \lambda -(1+u)c_{2})+c_{2}(\beta \lambda (-2u(u(2+7u(-1+\beta ))-7\beta )(u^{2}(-1+\beta )-\beta )+\beta (3\beta +u(-1+u+18\beta +u^{2}(17+3u-3(6+u)\beta )))\lambda -2(1+u(3+u))\beta^{2}\lambda^{2})+(9u{\left(u^{2}+\beta -u^{2}\beta \right)}^{2}+2(1+u(5+u))(u^{2}(-1+\beta )-\beta )\beta \lambda +(1+u(3+u))\beta^{2}\lambda^{2})c_{2})))/(\beta \lambda {\left(6u^{3}(-1+\beta )-6u\beta +(1+u(4+u))\beta \lambda \right)}^{2}\right)$

**Fig 4:**
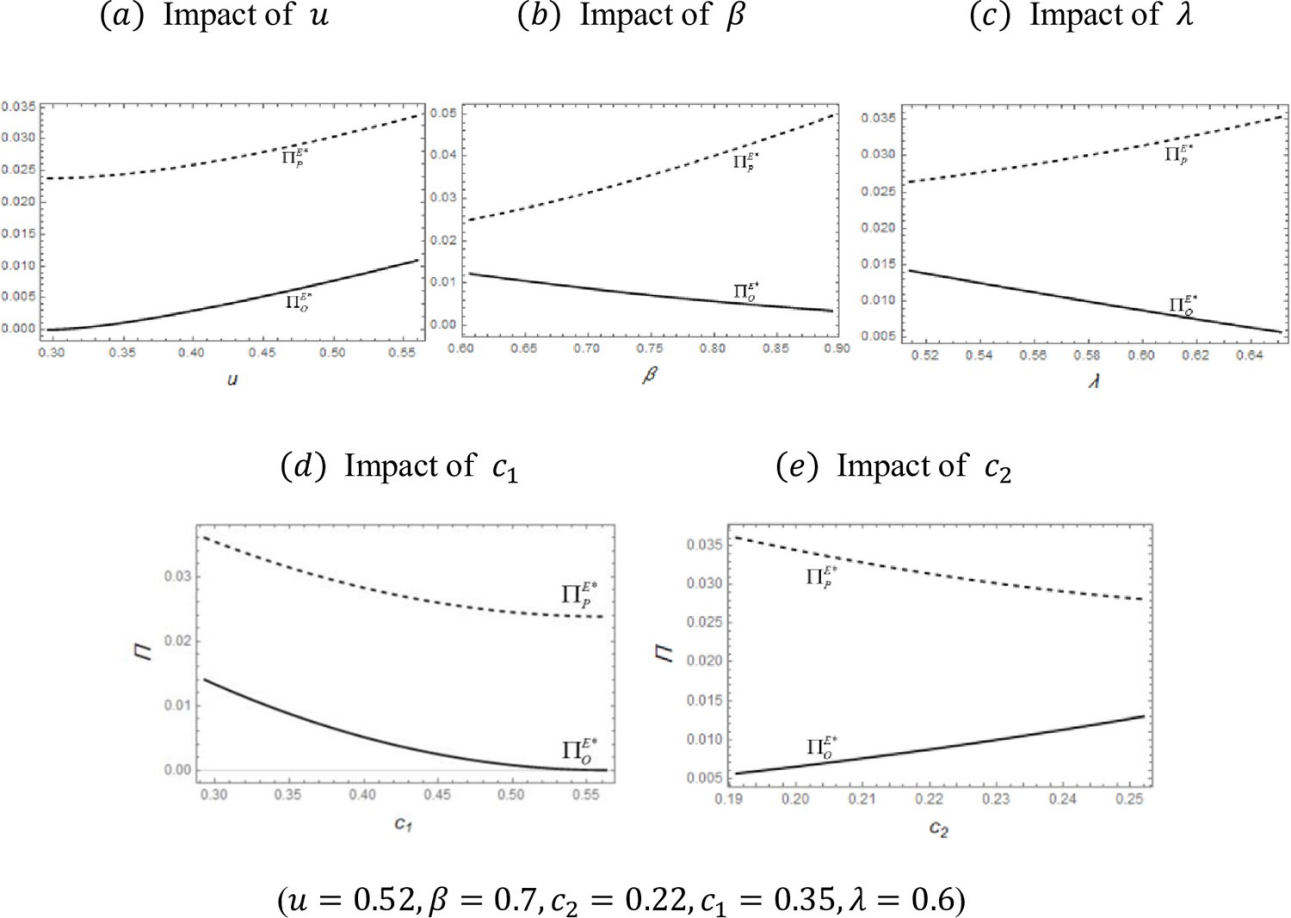
Impact of exogenous variables on $\Pi_{O}^{E*}\;and\;\Pi_{P}^{E*}$. (*a*) Impact of *u*, (*b*) Impact of *β*, (*c*) Impact of *λ*, (*d*) Impact of *c*_1_, (*e*) Impact of *c*_2_.

Lemma 4 shows that the equilibrium solution of the problem exists, so we can analyze the results under the equilibrium solution. Due to the complexity of the above results, we demonstrate their properties by numerical analysis, with the results shown in Fig 4.

Fig 4 shows the OEM and platform profit curves with varying parameters *u*, *β*, or *λ*. From Fig 4(A), we can easily find that both $\Pi_{O}^{E*}$ and $\Pi_{P}^{E*}$ increase as the usage level *u* increases. The reason the OEM’s profit $\Pi_{O}^{E*}$ rises is that as the product’s frequency of use increases, the Product 1 price and demand both increase. The reason for the rising platform profit $\Pi_{P}^{E*}$ is more complex. First, we analyze the gain from renting Product 2, which is declining. In detail, as the frequency of use increases, the consumers’ desire to rent decreases. To slow the decline in the rental demand for Product 2, its rental price decreases. Next, we analyze the commission income obtained by the Product 1 rental, which is rising. As the frequency of use increases, the increase in the Product 1 purchase volume drives down its rental price, which in turn drives the increase in its rental demand. Finally, we combine the above analyses to characterize the results of the struggle between the incomes of Products 1 and 2; the former is higher than the latter, so the platform’s income shows an upward trend.

Fig 4(B) shows that as the rental discount *β* increases, the platform profit $\Pi_{P}^{E*}$ increases and the OEM profit $\Pi_{O}^{E*}$ decreases. The reason for the reduced OEM profit $\Pi_{O}^{E*}$ is that as the frequency of use by consumers increases, Product 1’s price rises and the demand for it increases. As the consumers’ perception of the value of rented products increases, the demand for purchased products declines. To slow down the downward trend in demand, the Product 1 price decreases. To better illustrate this phenomenon, we divide the analysis of platform profit $\Pi_{P}^{E*}$ into two parts. First, we analyze the gain from renting Product 2, which is higher. We see that as the rental discount increases, consumer desire to rent increases. The Product 2 rental demand increases, so its rental price also increases. Next, we analyze the commission income obtained by Product 1 rentals, which first increases and then decreases. In detail, as the rental discount increases, the higher rental value of Product 1 causes fewer purchases of Product 1. Note that the rental price of Product 1 rises, and Product 1’s rent demand falls. When the rental discount is small, increasing the price has a greater impact on profit. Overall, the Product 1 rental income first increases and then decreases. Finally, we combine the above analysis to characterize the struggle between the incomes of Products 1 and 2; the latter is higher than the former, so the income of the platform rises.

Fig 4(C) shows that as the Product 2 quality *λ* increases, the platform profit $\Pi_{P}^{E*}$ increases and the OEM profit $\Pi_{O}^{E*}$ decreases. The reason for the OEM profit $\Pi_{O}^{E*}$ drop is that as Product 2’s quality increases, the Product 1 price and demand both increase. Next, we analyze the reasons for the change in platform profit $\Pi_{P}^{E*}$; as before, we look at it from two aspects. First, we analyze the gain from renting Product 2, which is up. Specifically, as the Product 2 quality increases, its rental demand increases, and its rental price also increases. Second, we analyze the revenue portion from renting Product 1, which is declining. In detail, as the quality of Product 1’s competing products increases, the rental demand for Product 1 decreases. Because Product 1 competes with the platform’s product, the rental service fee set by the platform increases. The increase in fees is lower than the decrease in demand. To finish, we combine the above analyses to characterize the outcome of the struggle between the incomes of Products 1 and 2; the latter is higher than the former, so the platform’s income rises.

Fig 4(D) shows that as Product 1’s cost *c*_1_ increases, the platform profit $\Pi_{P}^{E*}$ and OEM profit $\Pi_{O}^{E*}$ decrease. The reason for the OEM profit $\Pi_{O}^{E*}$ drop is that as Product 1’s cost increases, Product 1’s price increases, and this reduces the demand for buying Product 1. Next, we analyze the reasons for the change in platform profit $\Pi_{P}^{E*}$; as before, we look at it from two aspects. First, we analyze the gain from renting Product 2, which is up. Specifically, as the Product 1 cost increases, its rental demand increases, and its rental price also increases. Second, we analyze the revenue portion from renting Product 1, which is declining. In detail, as the cost of Product 1 increases, the rental demand for Product 1 and the fee both decrease. Overall, the total decrease in incomes from Product 1 is higher than the total increase in income from Product 2, so the platform’s income falls.

Fig 4(E) shows that as Product 2’s cost *c*_2_ increases, the platform profit $\Pi_{P}^{E*}$ decreases and the OEM profit $\Pi_{O}^{E*}$ increases. The reason the OEM profit $\Pi_{O}^{E*}$ increases is that as Product 2’s cost increases, the Product 1 price and demand both increase. Next, we analyze the reasons for the change in platform profit $\Pi_{P}^{E*}$; as before, we look at it from two aspects. First, we analyze the gain from renting Product 2, which is down. Specifically, as Product 2’s cost increases, its rental price increases, which leads to its rental demand decreasing. Second, we analyze the revenue portion from renting Product 1, which is up. In detail, as the cost of Product 1 increases, the rental demand for Product 1 increases. The rental service fee set by the platform decreases. The decrease in fees is lower than the increase in demand. Overall, the decrease in incomes of Product 1 is higher than the increase in income of Product 2, so the platform’s income declines.

In the next subsection, we compare the equilibrium results in the previous two subsections to gain managerial implications for platform mode selection.

### 5.3 Comparative analysis

**Fig 5:**
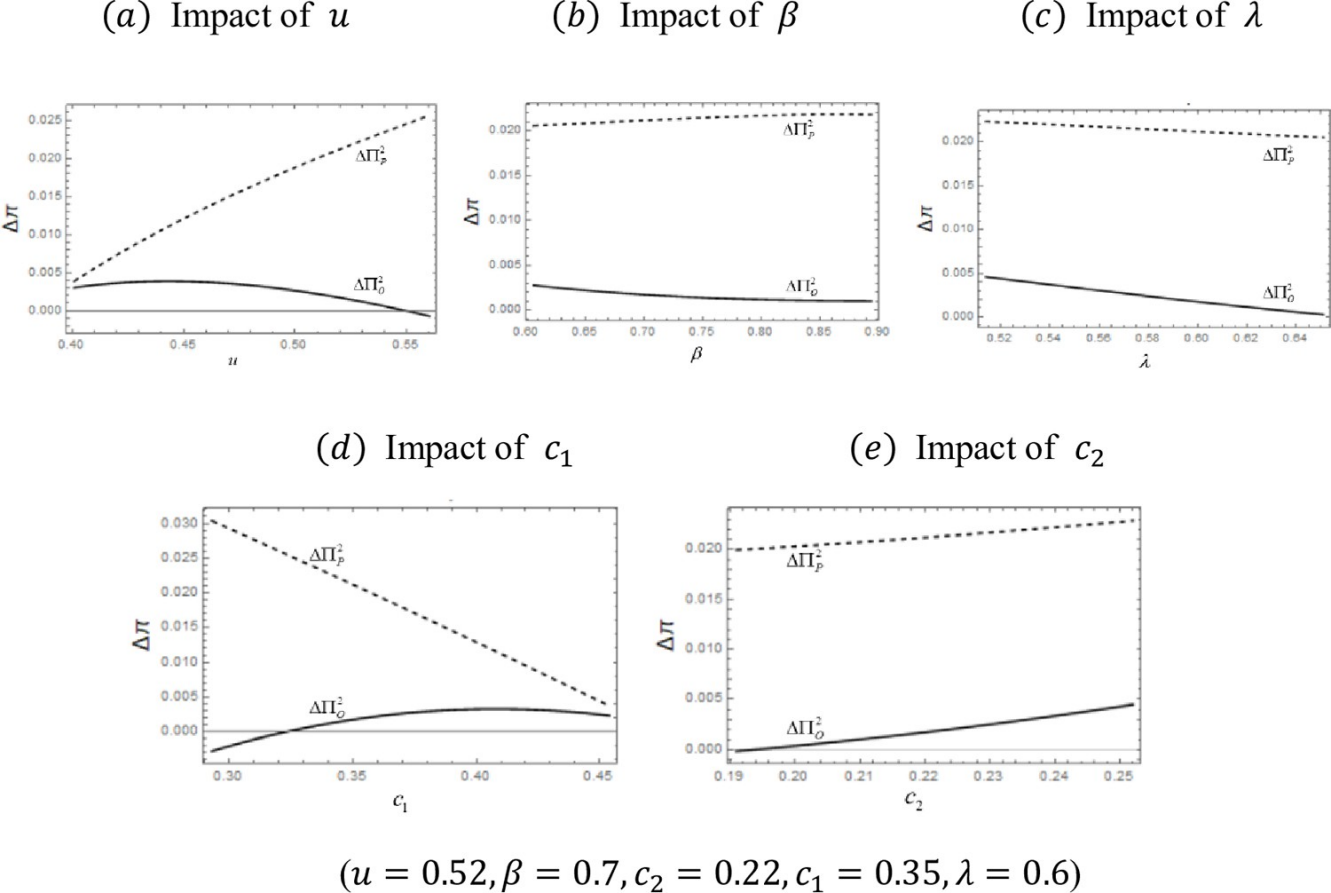
Impact of exogenous variables on $\Delta \Pi_{O}^{2}$ and $\Delta \Pi_{P}^{2}.$. (*a*) Impact of *u*, (*b*) Impact of *β*, (*c*) Impact of *λ*, (*d*), Impact of *c*_1_, (*e*) Impact of *c*_2_.

We compare the two model cases in which the platform decides the rental price of Product 2 by considering the profit difference. Let $\Delta \Pi_{O}^{2}$ denote the difference in the OEM profits $\Pi_{O}^{E*}-\Pi_{O}^{D*}$ and $\Delta \Pi_{P}^{2}$ denote the difference in platform profits $\Pi_{P}^{E*}-\Pi_{P}^{D*}$. Since the expressions of profit are quite complicated, we use a set of typical values to analyze the properties of the two modes; Fig 5 shows the results.

Fig 5 shows the profit curves as parameters *u*, *β*, *λ*, *c*_1_, and *c*_2_ vary. When the curve is over the abscissa, the margin is positive, and the OEM or platform prefer the hybrid sharing mode. From Fig 5, we can see that $\Delta \Pi_{P}^{2}$ decreases in the usage level *u*, whereas $\Delta \Pi_{O}^{2}$ increases. Also, $\Delta \Pi_{P}^{2}$ decreases in rental discount *β*, whereas $\Delta \Pi_{O}^{2}$ increases in rental discount *β*. We note that $\Delta \Pi_{P}^{2}$ and $\Delta \Pi_{O}^{2}$ both decrease in Product 2’s quality *λ*. Moreover, $\Delta \Pi_{P}^{2}$ decreases in Product 1’s cost *c*_1_, whereas $\Delta \Pi_{O}^{2}$ increases. Also, $\Delta \Pi_{P}^{2}$ and $\Delta \Pi_{O}^{2}$ increase in Product 2’s cost *c*_2_.

An important implication from the above analysis is that P2P sharing can result in higher OEM profit when the consumer usage level is low. Our findings indicate that when the rental discount is smaller, adding P2P sharing benefits the B2C platform more. Our findings also indicate that the sharing platform always benefits from hybrid sharing, and the platform benefits more from adding P2P sharing when the quality difference between the platform’s product and the OEM’s product is larger.

### 6. Conclusion

The sharing economy is booming in the fields of travel, residences, and clothing. Some platforms use the B2C mode, such as Hello-bike, and some use the P2P mode, such as Uber. There are also platforms employing the hybrid mode, such as Didi. Our research compares the B2C mode and hybrid mode, examining the differences in optimal decision-making and profits of the OEM and platform.

Our research reveals important and interesting results. First, introducing P2P service always benefits the B2C platform but sometimes harms the OEM. When consumer usage is high, introducing P2P sharing service will harm the OEM’s profits. Second, the profits of the platform and OEM are significantly affected by the usage level of consumers, quality differences of market products, and consumers’ rental discounts for the valuation of rented products. The platform’s optimal choice between B2C mode or hybrid mode also depends on changes in these factors. Specifically, when the usage level is low, the consumer rental discount is large, and the quality of the two products is close, the OEM can obtain higher income using B2C mode. When the usage level is high, the rental discount is small, and the quality difference between the two products is great, the OEM profit will be damaged by switching to hybrid mode. This finding may explain why the Didi platform uses the hybrid mode: handling requests for both personal vehicles and platform vehicles: the quality of platform vehicles and personal vehicles is relatively close, consumers have a good experience when riding a rental vehicle, and the frequency of use of such vehicles is not high. Third, in some cases, compared to in B2C mode, consumers have increased purchasing demand for the OEM’s product in hybrid mode. Specifically, that demand increases when the usage level is low, the rental discount is large, and the differences in product quality are small. Finally, our result shows that when the platform can set the rental price, the platform is more inclined to choose the hybrid mode, and the OEM will get more profit in hybrid mode.

While our research provides managerial implications for OEMs and platforms, certain limitations suggest further study. First, our research does not consider competition among multiple platforms. For example, three platforms currently provide sharing of bicycles in China: Hello-bike, Meituan Bike, and Didi Bike. Whether the choice of mode makes a difference in this competitive context is a question well worth investigating. Moreover, our research mainly aims at the comparison of B2C mode and hybrid mode. If we study these issues from the perspective of P2P mode and hybrid mode, there may be different findings. Whether the findings are analogous or dissimilar, they would have interesting practical implications.

## Supporting information

S1 Appendix (DOCX)
