# Supplementary material for: Introducing P2P service to a B2C sharing platform: A hybrid sharing mode
Source: PLoS One. 2022 Dec 30;17(12):e0279615. doi: 10.1371/journal.pone.0279615 (PMC9803320; doi:10.1371/journal.pone.0279615)
Supplement: S1 Appendix — (DOCX) [file pone.0279615.s001.docx]

## Appendix

**Proof of Lemma 1.** This lemma states that the problem is convex and has an optimal solution. It gives the value of the equilibrium solution and brings it into the original formula to get the demand and profit at equilibrium.

Consider $\pi_{O}^{N}(p)^{''}=-\frac{2}{u\left( 1-\beta\lambda\right)}<0$. The second derivative of this profit function is less than 0, and it has a maximum value. By the first-order condition $\pi_{O}^{N}(p)^{'}=\frac{-2p+u-u\beta\lambda+c_{1}+ur_{2}}{u\left( 1-\beta\lambda\right)}=0$, we get $p^{N*}=\frac{c_{1}+u\left( 1-\beta\lambda+r_{2} \right)}{2}$.

**Proof of Proposition 1.** This proposition is obtained by our sensitivity analysis of the results in Lemma 1.

(1) Sensitivity analysis of consumer usage levels ($u$)

$\frac{dp^{N*}}{du}=\frac{1-\beta\lambda+r_{2}}{2}>0$, $\frac{dD_{1}^{NB*}}{du}=\frac{c_{1}}{2u^{2}(1-\beta\lambda)}>0$, $\frac{dD_{2}^{NR*}}{du}=\frac{c_{1}}{2u^{2}\left( -1+\beta\lambda\right)}<0$, $\frac{d\Pi_{P}^{N*}}{du}=-\frac{c_{1}\left( r_{2}-c_{2} \right)}{2u^{2}\left( 1-\beta\lambda\right)}<0$, and $\frac{d\Pi_{O}^{N*}}{du}=\frac{{c_{1}}^{2}-u^{2}\left( 1-\beta\lambda+r_{2} \right)^{2}}{4u^{2}\left( -1+\beta\lambda\right)}>0$.

(2) Sensitivity analysis of perceived value of rental products ($\beta$)

$\frac{dp^{N*}}{d\beta}=-\frac{u\lambda}{2}<0$. $\frac{dD_{1}^{NB*}}{d\beta}=\frac{\lambda\left( -c_{1}+ur_{2} \right)}{2u\left( 1-\beta\lambda\right)^{2}}$, so if $c_{1}<ur_{2}$, then $\frac{dD_{1}^{NB*}}{d\beta}>0$, but if $c_{1}>ur_{2}$, then $\frac{dD_{1}^{NB*}}{d\beta}<0$.

$\frac{dD_{2}^{NR*}}{d\beta}=\frac{\beta^{2}\lambda^{2}c_{1}+u\left( 2+\beta\lambda\left( -4+\beta\lambda\right) \right)r_{2}}{2u\beta^{2}\lambda\left( 1-\beta\lambda\right)^{2}}$, so if $c_{1}>\frac{-u\left( 2+\beta\lambda\left( -4+\beta\lambda\right) \right)r_{2}}{\beta^{2}\lambda^{2}}$, then $\frac{dD_{2}^{NR*}}{d\beta}>0$, but if $c_{1}<\frac{-u\left( 2+\beta\lambda\left( -4+\beta\lambda\right) \right)r_{2}}{\beta^{2}\lambda^{2}}$, then $\frac{dD_{2}^{NR*}}{d\beta}<0$.

$\frac{d\Pi_{O}^{N*}}{d\beta}=\frac{\lambda\left( c_{1}+u\left( -1+\beta\lambda-r_{2} \right) \right)\left( c_{1}+u\left( 1-\beta\lambda-r_{2} \right) \right)}{4u\left( 1-\beta\lambda\right)^{2}}$, so if $c_{1}>u(r_{2}+1-\beta\lambda)$ or $c_{1}<u(r_{2}-1+\beta\lambda)$, then $\frac{d\Pi_{O}^{N*}}{d\beta}>0$, but if $u(r_{2}-1+\beta\lambda)<c_{1}<u(r_{2}+1-\beta\lambda)$, then $\frac{d\Pi_{O}^{N*}}{d\beta}<0$.

$\frac{d\Pi_{P}^{N*}}{d\beta}=\frac{\left( r_{2}-c_{2} \right)\left( \beta^{2}\lambda^{2}c_{1}+u\left( 2+\beta\lambda\left( -4+\beta\lambda\right) \right)r_{2} \right)}{2u\beta^{2}\lambda\left( -1+\beta\lambda\right)^{2}}$, so if $c_{1}>\frac{-u\left( 2+\beta\lambda\left( -4+\beta\lambda\right) \right)r_{2}}{\beta^{2}\lambda^{2}}$, then $\frac{d\Pi_{P}^{N*}}{d\beta}>0$, but if $c_{1}<\frac{-u\left( 2+\beta\lambda\left( -4+\beta\lambda\right) \right)r_{2}}{\beta^{2}\lambda^{2}}$, then $\frac{d\Pi_{P}^{N*}}{d\beta}<0$.

(3) Sensitivity analysis of Product 2 quality ($\lambda$)

$\frac{dp^{N*}}{d\lambda}=-\frac{u\beta}{2}<0$. $\frac{dD_{1}^{NB*}}{d\lambda}=\frac{\beta\left( -c_{1}+ur_{2} \right)}{2u\left( 1-\beta\lambda\right)^{2}}$, so if $c_{1}<ur_{2}$, then $\frac{dD_{1}^{NB*}}{d\lambda}>0$, but if $c_{1}>ur_{2}$, then $\frac{dD_{1}^{NB*}}{d\lambda}<0$.

$\frac{dD_{2}^{NR*}}{d\lambda}=\frac{\beta^{2}\lambda^{2}c_{1}+u\left( 2+\beta\lambda\left( -4+\beta\lambda\right) \right)r_{2}}{2u\beta\lambda^{2}\left( 1-\beta\lambda\right)^{2}}$, so if $c_{1}>\frac{-u\left( 2+\beta\lambda\left( -4+\beta\lambda\right) \right)r_{2}}{\beta^{2}\lambda^{2}}$, then $\frac{dD_{2}^{NR*}}{d\lambda}>0$, but if $c_{1}<\frac{-u\left( 2+\beta\lambda\left( -4+\beta\lambda\right) \right)r_{2}}{\beta^{2}\lambda^{2}}$, then $\frac{dD_{2}^{NR*}}{d\lambda}<0$.

$\frac{d\Pi_{O}^{N*}}{d\lambda}=\frac{\beta\left( c_{1}+u\left( -1+\beta\lambda-r_{2} \right) \right)\left( c_{1}+u\left( 1-\beta\lambda-r_{2} \right) \right)}{4u\left( 1-\beta\lambda\right)^{2}}$, so if $c_{1}>u(r_{2}+1-\beta\lambda)$ or $c_{1}<u(r_{2}-1+\beta\lambda)$, then $\frac{d\Pi_{O}^{N*}}{d\lambda}>0$, but if $u(r_{2}-1+\beta\lambda)<c_{1}<u(r_{2}+1-\beta\lambda)$, then $\frac{d\Pi_{O}^{N*}}{d\lambda}<0$.

$\frac{d\Pi_{P}^{N*}}{d\lambda}=\frac{\left( r_{2}-c_{2} \right)\left( \beta^{2}\lambda^{2}c_{1}+u\left( 2+\beta\lambda\left( -4+\beta\lambda\right) \right)r_{2} \right)}{2u\beta\lambda^{2}\left( 1-\beta\lambda\right)^{2}}$, so if $c_{1}>\frac{-u\left( 2+\beta\lambda\left( -4+\beta\lambda\right) \right)r_{2}}{\beta^{2}\lambda^{2}}$, then $\frac{d\Pi_{P}^{N*}}{d\lambda}>0$; but if $c_{1}<\frac{-u\left( 2+\beta\lambda\left( -4+\beta\lambda\right) \right)r_{2}}{\beta^{2}\lambda^{2}}$, then $\frac{d\Pi_{P}^{N*}}{d\lambda}<0$.

**Proof of Lemma 2.** This lemma states that the problem is convex and has an optimal solution. It gives the value of the equilibrium solution and brings it into the original formula to get the demand and profit at equilibrium.

$$H_{O}^{P}=\left( \begin{matrix} \frac{\partial^{2}\pi_{O}^{P}}{\partial p^{2}} & \frac{\partial^{2}\pi_{O}^{P}}{\partial p\partial t} \\ \frac{\partial^{2}\pi_{O}^{P}}{\partial t\partial p} & \frac{\partial^{2}\pi_{O}^{P}}{\partial t^{2}} \end{matrix} \right)=\left( \begin{matrix} \frac{2u}{u^{2}\left( -1+\beta\right)+\beta\left( -1+\lambda\right)} & \frac{\left( 1-u \right)u}{u^{2}\left( -1+\beta\right)+\beta\left( -1+\lambda\right)} \\ \frac{\left( 1-u \right)u}{u^{2}\left( -1+\beta\right)+\beta\left( -1+\lambda\right)} & 0 \end{matrix} \right)<0$$

$$H_{P}^{P}=\left( \begin{matrix} \frac{\partial^{2}\pi_{P}^{P}}{\partial p^{2}} & \frac{\partial^{2}\pi_{P}^{P}}{\partial p\partial t} \\ \frac{\partial^{2}\pi_{P}^{P}}{\partial t\partial p} & \frac{\partial^{2}\pi_{P}^{P}}{\partial t^{2}} \end{matrix} \right)=\left( \begin{matrix} 0 & -\frac{\left( -1+u \right)u}{u^{2}\left( -1+\beta\right)+\beta\left( -1+\lambda\right)} \\ -\frac{\left( -1+u \right)u}{u^{2}\left( -1+\beta\right)+\beta\left( -1+\lambda\right)} & \frac{2\left( -1+u \right)^{2}u}{u^{2}\left( -1+\beta\right)+\beta\left( -1+\lambda\right)} \end{matrix} \right)<0$$

The Hessian matrix of this profit function is less than 0, and it has a maximum value. By the first-order conditions $\pi_{O}^{P}(p)^{'}=-\frac{u\left( -2p+t\left( -1+u \right)+u+\beta-\beta\left( u+\lambda\right)+c_{1}+r_{2} \right)}{u^{2}\left( -1+\beta\right)+\beta\left( -1+\lambda\right)}=0$ and $\pi_{P}^{P}(t)^{'}=\frac{\left( -1+u \right)\left( u\left( -p+2t\left( -1+u \right)+u+\beta-\beta\left( u+\lambda\right) \right)-c_{2}+\left( 1+u \right)r_{2} \right)}{u^{2}\left( -1+\beta\right)+\beta\left( -1+\lambda\right)}=0$, we get $p^{P*}=\frac{u\left( u+\beta-u\beta-\beta\lambda\right)+2uc_{1}+c_{2}+\left( -1+u \right)r_{2}}{3u}$ and $t^{P*}=\frac{u\left( u\left( -1+\beta\right)+\beta\left( -1+\lambda\right) \right)+uc_{1}+2c_{2}-\left( 2+u \right)r_{2}}{3\left( -1+u \right)u}$.

**Proof of Proposition 2.** This proposition is obtained by our sensitivity analysis of the results in Lemma 2.

(1) Sensitivity analysis of consumer usage levels (*u*)

$\frac{dp^{P*}}{du}=\frac{u^{2}\left( 1-\beta\right)+r_{2}-c_{2}}{3u^{2}}>0.$ $\frac{dt^{P*}}{du}=\frac{u^{2}\left( 1-\beta\lambda\right)+\left( 2-4u \right)(c_{2}-r_{2})+u^{2}(r_{2}-c_{1})}{3\left( -1+u \right)^{2}u^{2}}$, so if $u^{2}\left( 1-\beta\lambda\right)+\left( 2-4u \right)(c_{2}-r_{2})+u^{2}(r_{2}-c_{1})>0$, then $\frac{dt^{P*}}{du}>0$, but if $u^{2}\left( 1-\beta\lambda\right)+\left( 2-4u \right)(c_{2}-r_{2})+u^{2}(r_{2}-c_{1})<0$, then $\frac{dt^{P*}}{du}<0$.

(2) Sensitivity analysis of perceived value of rental products ($\beta$)

$\frac{dp^{P*}}{d\beta}=\frac{1}{3}\left( 1-u-\lambda\right)$, $\frac{dt^{P*}}{d\beta}=\frac{-1+u+\lambda}{3\left( -1+u \right)}$, so if $u<1-\lambda$，then $\frac{dp^{P*}}{d\beta}>0$ and $\frac{dt^{P*}}{d\beta}>0$, but if $u>1-\lambda$, then $\frac{dp^{P*}}{d\beta}<0$ and $\frac{dt^{P*}}{d\beta}<0$.

(3) Sensitivity analysis of Product 2 quality ($\lambda$)

$\frac{dp^{P*}}{d\lambda}=-\frac{\beta}{3}<0$ and $\frac{dt^{P*}}{d\lambda}=\frac{\beta}{3\left( -1+u \right)}<0$.

**Proof of Lemma 3.** This lemma states that the problem is convex and has an optimal solution. It gives the value of the equilibrium solution and brings it into the original formula to get the demand and profit at equilibrium.

$$H_{O}^{D}=\left( \begin{matrix} \frac{\partial^{2}\pi_{O}^{D}}{\partial p^{2}} & \frac{\partial^{2}\pi_{O}^{D}}{\partial p\partial r_{2}} \\ \frac{\partial^{2}\pi_{O}^{D}}{\partial r_{2}\partial p} & \frac{\partial^{2}\pi_{O}^{D}}{\partial{r_{2}}^{2}} \end{matrix} \right)=\left( \begin{matrix} -\frac{2}{u-u\beta\lambda} & \frac{1}{1-\beta\lambda} \\ \frac{1}{1-\beta\lambda} & 0 \end{matrix} \right)<0$$

$$H_{P}^{D}=\left( \begin{matrix} \frac{\partial^{2}\pi_{P}^{D}}{\partial p^{2}} & \frac{\partial^{2}\pi_{P}^{D}}{\partial p\partial r_{2}} \\ \frac{\partial^{2}\pi_{P}^{D}}{\partial r_{2}\partial p} & \frac{\partial^{2}\pi_{P}^{D}}{\partial{r_{2}}^{2}} \end{matrix} \right)=\left( \begin{matrix} 0 & \frac{1}{u-u\beta\lambda} \\ \frac{1}{u-u\beta\lambda} & -\frac{2}{\beta\lambda-\beta^{2}\lambda^{2}} \end{matrix} \right)<0$$

The Hessian matrix of this profit function is less than 0, and it has a maximum value. By the first-order conditions $\pi_{O}^{D}(p)^{'}=-\frac{u\left( -2p+t\left( -1+u \right)+u+\beta-\beta\left( u+\lambda\right)+c_{1}+r_{2} \right)}{u^{2}\left( -1+\beta\right)+\beta\left( -1+\lambda\right)}=0$ and $\pi_{P}^{D}(t)^{'}=\frac{\left( -p+\left( -1+u \right)\left( t\left( 1+u \right)+u\left( -1+\beta\right) \right) \right)\beta\lambda+\left( u^{2}\left( -1+\beta\right)-\beta\right)\left( c_{2}-2r_{2} \right)}{\beta\left( u^{2}\left( -1+\beta\right)+\beta\left( -1+\lambda\right) \right)\lambda}=0$, we get $p^{D*}=-\frac{u\left( -2p+t\left( -1+u \right)+u+\beta-\beta\left( u+\lambda\right)+c_{1}+r_{2} \right)}{u^{2}\left( -1+\beta\right)+\beta\left( -1+\lambda\right)}$ and ${t^{D}}^{*}=\frac{\left( -1+u \right)\left( u\left( -p+2t\left( -1+u \right)+u+\beta-\beta\left( u+\lambda\right) \right)-c_{2}+\left( 1+u \right)r_{2} \right)}{u^{2}\left( -1+\beta\right)+\beta\left( -1+\lambda\right)}$.

**Proof of Proposition 3.** This proposition is obtained by our sensitivity analysis of the results in Lemma 3.

(1) Sensitivity analysis of consumer usage levels (*u*)

$\frac{dp^{D*}}{du}=-\frac{2-2\beta\lambda+c_{2}}{-4+\beta\lambda}>0$, $\frac{dr_{2}^{D*}}{du}=\frac{\beta\lambda c_{1}}{u^{2}\left( -4+\beta\lambda\right)}<0$, $\frac{dD_{1}^{DB*}}{du}=\frac{\left( 2-\beta\lambda\right)c_{1}}{u^{2}\left( -4+\beta\lambda\right)\left( -1+\beta\lambda\right)}>0$, $\frac{dD_{2}^{DR*}}{du}=-\frac{c_{1}}{u^{2}\left( 4-5\beta\lambda+\beta^{2}\lambda^{2} \right)}<0$, $\frac{d\Pi_{O}^{D*}}{du}=\frac{\left( \left( 2-\beta\lambda\right)c_{1}+u\left( -2+2\beta\lambda-c_{2} \right) \right)\left( \left( -2+\beta\lambda\right)c_{1}+u\left( -2+2\beta\lambda-c_{2} \right) \right)}{u^{2}\left( -4+\beta\lambda\right)^{2}\left( 1-\beta\lambda\right)}>0$, $\frac{d\Pi_{P}^{D*}}{du}=\frac{2c_{1}\left( \beta\lambda\left( u-u\beta\lambda+c_{1} \right)+u\left( -2+\beta\lambda\right)c_{2} \right)}{u^{3}\left( -4+\beta\lambda\right)^{2}\left( -1+\beta\lambda\right)}<0$.

(2) Sensitivity analysis of perceived value of rental products ($\beta$)

$\frac{dp^{D*}}{d\beta}=\frac{\lambda\left( 2c_{1}+u\left( -6+c_{2} \right) \right)}{\left( -4+\beta\lambda\right)^{2}}<0$. $\frac{dr_{2}^{D*}}{d\beta}=\frac{\lambda\left( 4c_{1}+u\left( 4+\beta\lambda\left( -8+\beta\lambda\right)+2c_{2} \right) \right)}{u\left( -4+\beta\lambda\right)^{2}}$, so if $c_{1}>\frac{-u\left( 4+\beta\lambda\left( -8+\beta\lambda\right)+2c_{2} \right)}{4}$, then $\frac{dr_{2}^{D*}}{d\beta}>0$, but if $c_{1}<\frac{-u\left( 4+\beta\lambda\left( -8+\beta\lambda\right)+2c_{2} \right)}{4}$, then $\frac{dr_{2}^{D*}}{d\beta}<0$.

(3) Sensitivity analysis of Product 2 quality ($\lambda$)

$\frac{dp^{D*}}{d\lambda}=\frac{\beta\left( 2c_{1}+u\left( -6+c_{2} \right) \right)}{\left( -4+\beta\lambda\right)^{2}}<0$. $\frac{dr_{2}^{D*}}{d\lambda}=\frac{\beta\left( 4c_{1}+u\left( 4+\beta\lambda\left( -8+\beta\lambda\right)+2c_{2} \right) \right)}{u\left( -4+\beta\lambda\right)^{2}}$, so if $c_{1}>\frac{-u\left( 4+\beta\lambda\left( -8+\beta\lambda\right)+2c_{2} \right)}{4}$, then $\frac{dr_{2}^{D*}}{d\lambda}>0$, but if $c_{1}<\frac{-u\left( 4+\beta\lambda\left( -8+\beta\lambda\right)+2c_{2} \right)}{4}$, then $\frac{dr_{2}^{D*}}{d\lambda}<0$.

(4) Sensitivity analysis of Product 1’s cost ($c_{1}$)

$\frac{dp^{D*}}{dc_{1}}=\frac{2}{4-\beta\lambda}>0$, $\frac{dr_{2}^{D*}}{dc_{1}}=\frac{\beta\lambda}{4u-u\beta\lambda}>0$, $\frac{dD_{1}^{DB*}}{dc_{1}}=\frac{-2+\beta\lambda}{u(4-5\beta\lambda+\beta^{2}\lambda^{2})}<0$, $\frac{dD_{2}^{DR*}}{dc_{1}}=\frac{1}{u(4-5\beta\lambda+\beta^{2}\lambda^{2})}>0$, $\frac{d\Pi_{O}^{D*}}{dc_{1}}=\frac{2(-2+\beta\lambda)((-2+\beta\lambda)c_{1}+u(2-2\beta\lambda+c_{2}))}{u\left( -4+\beta\lambda\right)^{2}(1-\beta\lambda)}<0$, $\frac{d\Pi_{P}^{D*}}{dc_{1}}=\frac{2(\beta\lambda(u-u\beta\lambda+c_{1})+u(-2+\beta\lambda)c_{2})}{u^{2}\left( -4+\beta\lambda\right)^{2}(1-\beta\lambda)}>0$.

(5) Sensitivity analysis of Product 2’s cost ($c_{2}$)

$\frac{dp^{D*}}{dc_{2}}=\frac{u}{4-\beta\lambda}>0$, $\frac{dr_{2}^{D*}}{dc_{2}}=\frac{2}{4-\beta\lambda}>0$, $\frac{dD_{1}^{DB*}}{dc_{2}}=\frac{1}{4-5\beta\lambda+\beta^{2}\lambda^{2}}>0$, $\frac{dD_{2}^{DR*}}{dc_{2}}=\frac{-2+\beta\lambda}{\beta\lambda(4-5\beta\lambda+\beta^{2}\lambda^{2})}<0$, $\frac{d\Pi_{O}^{D*}}{dc_{2}}=\frac{2((-2+\beta\lambda)c_{1}+u(2-2\beta\lambda+c_{2}))}{\left( -4+\beta\lambda\right)^{2}(1-\beta\lambda)}>0$, $\frac{d\Pi_{P}^{D*}}{dc_{2}}=\frac{2(-2+\beta\lambda)(\beta\lambda(u-u\beta\lambda+c_{1})+u(-2+\beta\lambda)c_{2})}{u\beta\lambda\left( -4+\beta\lambda\right)^{2}(1-\beta\lambda)}<0$.

**Proof of Lemma 4.** This lemma states that the problem is convex and has an optimal solution. It gives the value of the equilibrium solution and brings it into the original formula to get the demand and profit at equilibrium.

$$H_{O}^{E}=\left( \begin{matrix} \frac{\partial^{2}\pi_{O}^{E}}{\partial p^{2}} & \frac{\partial^{2}\pi_{O}^{E}}{\partial p\partial r_{2}} & \frac{\partial^{2}\pi_{O}^{E}}{\partial p\partial t} \\ \frac{\partial^{2}\pi_{O}^{E}}{\partial r_{2}\partial p} & \frac{\partial^{2}\pi_{O}^{E}}{\partial{r_{2}}^{2}} & \frac{\partial^{2}\pi_{O}^{E}}{\partial r_{2}\partial t} \\ \frac{\partial^{2}\pi_{O}^{E}}{\partial t\partial p} & \frac{\partial^{2}\pi_{O}^{E}}{\partial t\partial r_{2}} & \frac{\partial^{2}\pi_{O}^{E}}{\partial t^{2}} \end{matrix} \right)=\left( \begin{matrix} \frac{2}{u\left( -1+\beta\right)} & 0 & \frac{1-u}{u\left( -1+\beta\right)} \\ 0 & 0 & 0 \\ \frac{1-u}{u\left( -1+\beta\right)} & 0 & 0 \end{matrix} \right)<0$$

$$H_{P}^{E}=\left( \begin{matrix} \frac{\partial^{2}\pi_{P}^{E}}{\partial p^{2}} & \frac{\partial^{2}\pi_{P}^{E}}{\partial p\partial r_{2}} & \frac{\partial^{2}\pi_{P}^{E}}{\partial p\partial t} \\ \frac{\partial^{2}\pi_{P}^{E}}{\partial r_{2}\partial p} & \frac{\partial^{2}\pi_{P}^{E}}{\partial{r_{2}}^{2}} & \frac{\partial^{2}\pi_{P}^{E}}{\partial r_{2}\partial t} \\ \frac{\partial^{2}\pi_{P}^{E}}{\partial t\partial p} & \frac{\partial^{2}\pi_{P}^{E}}{\partial t\partial r_{2}} & \frac{\partial^{2}\pi_{P}^{E}}{\partial t^{2}} \end{matrix} \right)=\left( \begin{matrix} 0 & 0 & \frac{1}{1-\beta} \\ 0 & -\frac{2}{\beta\lambda-\beta\lambda^{2}} & \frac{u}{\beta-\beta\lambda} \\ \frac{1}{1-\beta} & \frac{u}{\beta-\beta\lambda} & \frac{2\left( -1+u \right)}{-1+\beta} \end{matrix} \right)<0$$

The Hessian matrix of this profit function is less than 0, and it has a maximum value. By the first-order conditions $\pi_{O}^{E}(p)^{'}=-\frac{u\left( -2p+t\left( -1+u \right)+u+\beta-\beta\left( u+\lambda\right)+c_{1}+r_{2} \right)}{u^{2}\left( -1+\beta\right)+\beta\left( -1+\lambda\right)}=0$, $\pi_{P}^{E}(t)^{'}=\frac{\left( -1+u \right)\left( u\left( -p+2t\left( -1+u \right)+u+\beta-\beta\left( u+\lambda\right) \right)-c_{2}+\left( 1+u \right)r_{2} \right)}{u^{2}\left( -1+\beta\right)+\beta\left( -1+\lambda\right)}=0$ and $\pi_{P}^{E}(r_{2})^{'}=\frac{\left( -p+\left( -1+u \right)\left( t\left( 1+u \right)+u\left( -1+\beta\right) \right) \right)\beta\lambda+\left( u^{2}\left( -1+\beta\right)-\beta\right)\left( c_{2}-2r_{2} \right)}{\beta\left( u^{2}\left( -1+\beta\right)+\beta\left( -1+\lambda\right) \right)\lambda}=0$. we get $p^{E*}=\frac{\left( 4u^{3}\left( -1+\beta\right)-4u\beta+\left( 1+u \right)^{2}\beta\lambda\right)c_{1}-\left( u^{2}\left( -1+\beta\right)+\beta\left( -1+\lambda\right) \right)\left( 2u^{2}\left( -1+\beta\right)+u\beta\left( -2+\lambda\right)+\beta\lambda-\left( 1+u \right)c_{2} \right)}{6u^{3}\left( -1+\beta\right)-6u\beta+\left( 1+u\left( 4+u \right) \right)\beta\lambda}$, ${t^{E}}^{*}=\frac{2u\left( u^{2}\left( -1+\beta\right)-\beta\right)\left( -u+\left( -1+u \right)\beta\right)+\beta\left( u\left( -1+\left( -1+u \right)u\left( -1+\beta\right)-\beta\right)+\beta\right)\lambda-\beta^{2}\lambda^{2}+\left( 2u^{3}\left( -1+\beta\right)+u\beta\left( -2+\lambda\right)+\beta\lambda\right)c_{1}+\left( -\left( -2+u \right)\left( u^{2}\left( -1+\beta\right)-\beta\right)+\beta\lambda\right)c_{2}}{\left( -1+u \right)\left( 6u^{3}\left( -1+\beta\right)-6u\beta+\left( 1+u\left( 4+u \right) \right)\beta\lambda\right)},$ and ${r_{2}^{E}}^{*}=\frac{u\beta\lambda\left( u+4u^{2}\left( -1+\beta\right)+u\beta\left( -2+\lambda\right)+2\beta\left( -1+\lambda\right)+\left( -1+u \right)c_{1} \right)+\left( 3u^{3}\left( -1+\beta\right)+\beta\lambda+u\beta\left( -3+2\lambda\right) \right)c_{2}}{6u^{3}\left( -1+\beta\right)-6u\beta+\left( 1+u\left( 4+u \right) \right)\beta\lambda}$.
